# Simulation as a tool to model potential workflow enhancements in radiotherapy treatment pathways – A systematic review

**DOI:** 10.1002/acm2.14132

**Published:** 2023-09-03

**Authors:** Andrew Robinson, Md Asaduzzaman, Raj Jena, Roozbeh Naemi

**Affiliations:** ^1^ School of Health, Science and Wellbeing Staffordshire University Stoke on Trent UK; ^2^ Department of Medical Physics Cambridge University Hospitals NHS Foundation Trust Cambridge UK; ^3^ School of Digital Technologies and Arts Staffordshire University Stoke on Trent UK; ^4^ Department of Oncology University of Cambridge Cambridge UK

**Keywords:** computer simulations, discrete event simulation, operations research, pathway, radiotherapy, waiting times

## Abstract

This systematic review aimed to synthesize and summarize the use of simulation of radiotherapy pathways. The objective was to establish the suitability of those simulations in modeling the potential introduction of processes and technologies to speed up radiotherapy pathways. A systematic literature search was carried out using PubMed and Scopus databases to evaluate the use of simulation in radiotherapy pathways. Full journal articles and conference proceedings were considered, and the search was limited to the English language only. To be eligible for inclusion, articles had to model multiple sequential processes in the radiotherapy pathway concurrently to demonstrate the suitability of simulation modeling in typical pathways. Papers solely modeling scheduling, capacity, or queuing strategies were excluded. In total, 151 potential studies were identified and screened to find 18 relevant studies in October 2022. Studies showed that various pathways could be modeled, including the entire pathway from referral to end of treatment or the constituent phases such as pre‐treatment, treatment, or other subcomponents. The data required to generate models varied from study to study, but at least 3 months of data were needed. This review demonstrates that modeling and simulation of radiotherapy pathways are feasible and that model output matches real‐world systems. Validated models give researchers confidence to modify models with potential workflow enhancements to assess their potential effect on real‐world systems. It is recommended that researchers follow best practice guidelines when building models to ensure that they are fit for purpose and to enable decision makers to have confidence in their results.

## 1 BACKGROUND

Radiotherapy is the treatment of disease using ionizing radiation. In the UK, approximately 20% of patients diagnosed with cancer will receive radiotherapy as part of their treatment.^1^ Optimal utilization of radiotherapy varies by cancer type, but it is estimated to be at least 40% and potentially over 50%.^2^, ^3^ Some of this under‐utilization may be attributed to a perception that radiotherapy is not a modern cancer treatment,^4^ fears from media coverage of overexposures, and other misperceptions.^5^ By 2025 there would need to be around 211,000 courses of radiotherapy in the UK annually to reach optimal utilization.^3^

In the UK, the NHS constitution outlines operational standards for diagnosing and treating cancers following a referral for suspected cancer. These are expressed as percentages of patients that start treatment within 31 days from the decision to treat (DTT) or earliest clinically appropriate date (ECAD) of second or subsequent treatments and percentages of patients receiving first treatment within 62 days following an urgent referral for suspected cancer from a GP or NHS Cancer Screening Program.

Whilst compliance with national targets is highly desirable, the underlying reasons for complying with or performing better than these targets can have many facets. The time taken from DTT to treatment has been reviewed in the literature,^6^, ^7^, ^8^ suggesting poorer outcomes with the increased time between DTT and treatment.^7^ Meta‐analyses have shown a significant link between delay and the risk of local occurrence in head, neck, and breast cancer^9^; the authors of this study suggest that until further studies have been performed that it is assumed that a similar relationship would be observed for all cancer types. A more recent analysis by Hanna et al. across different clinical indications showed that for a 4‐week delay in cancer treatment, there is an increase in mortality of approximately 10%.^10^ Increasing the time between DTT and treatment may allow the spread of cancer beyond the original treatment volume (i.e., metastasise). However, it is difficult to model and quantify the increased risk of metastatic disease as a function of the time between DTT and treatment^6^ at both the patient and population levels. In addition to the potential clinical benefits of short waiting times (i.e., the patient's perceived waiting time from DTT to treatment), short waiting times for diagnosis and treatment are important to patients,^11^ with extended times potentially contributing to anxiety. Health systems are increasingly using quality indicators in radiation oncology, with waiting times being one of them, so waiting times should be as short as is reasonably achievable.^12^, ^13^

In the wake of the COVID‐19 pandemic, the UK's National Health Service (NHS) is under increased pressure due to cancer patients delaying their diagnosis and treatment during the various national lockdowns and restrictions. An estimated 40,000 fewer cases of cancer than anticipated were diagnosed in 2020,^14^ and estimates of years of life lost have been predicted to be in the order of 60,000 for patients with delayed diagnosis and treatment due to 12 months’ worth of physical distancing measures since March 2020.^15^ In the UK, radiotherapy fractions delivered have decreased by 11.4% compared to 2017,^16^ mainly due to the increased use of hypofractionation and a shift towards earlier diagnosis. A nationwide reduction in the number of treatment fractions would only directly ease issues associated with treatment machine capacity; radiotherapy instances still contribute to pre‐treatment resource utilization (such as CT imaging, clinical oncologist target definition, radiotherapy planning, etc.), even if the required treatment machine capacity has been reduced.

The radiotherapy pre‐treatment pathway from DTT to the patient starting treatment consists of many discrete tasks performed by multiple staff groups. Generally, each task depends on its predecessor and cannot be completed in parallel. These tasks include but are not limited to initial consultation and DTT, performing a CT scan and other secondary imaging that is used to identify what to treat and organs at risk (OARs), radiotherapy plan generation, plan authorization, plan checking, pre‐treatment patient specific quality control, and other pre‐treatment checks.

As with many scenarios in healthcare, waiting times can generally be reduced in two ways: either by utilizing additional resources (e.g., staff) or process changes that utilize better use of existing resources.^17^ Work has been performed on the capacity of departments to receive referrals and treatment machine capacity.^18^, ^19^ However, increasing treatment machine capacity (such as extending the operational time of linear accelerators) may not necessarily improve throughput time if there is a rate‐limiting step earlier in the process (such as treatment planning).

As such, and with the increasing financial strain on public health systems, any aid to improving capacity through the efficiency of radiotherapy treatment pathways should be explored. It was previously identified that operational research (OR) could be used for resource planning in radiotherapy in the following ways^20^:

- Patient scheduling: To maximize efficiencies through existing pathways through scheduling decisions.
- Strategic decision making: Exploring best practices to enhance the long‐term operation of a radiotherapy center.
- Resource capacity planning: Exploring ways to utilize existing resources.
- Patient prioritization: Optimizing access to patient groups whose disease benefits most from shorter access times.

It was also found that computer simulations (including Discrete Event Simulations [DES]) were the predominant methodology for strategic managerial decision‐making.^20^ Once a model of a system is built, a simulation can be run and compared against key performance indicators (KPIs) of a real system. If the model and real‐world KPIs are the same, decision‐makers can be confident that perturbations to the system can be accurately modeled. This allows so‐called “what‐if” scenarios to be performed in the model to find potential improvements in the real‐world system.

This review aimed to synthesize and summarize the use of computer simulation of radiotherapy pathways. The objective was to assess the suitability of simulation for determining the potential introduction of workflow enhancements to the radiotherapy pathway.

## 2 METHODS

### 2.1 Search strategy

A systematic review followed the Preferred Reporting Items for Systematic Reviews and Meta‐Analyses (PRISMA) guidelines.^21^ Searches were carried out using PubMed and Scopus databases. Full journal articles and the conference proceedings were considered; additional grey literature was not included as it does not necessarily meet the quality of peer‐reviewed literature.^22^ The search was limited to English, and no geographical restrictions were applied. The search was made on 23 October 2022. No limit was placed on the publication date. Manual searches of reference lists were also performed. Full search methodology, including database‐specific search terms and a PRISMA checklist, are available on reasonable request. Database‐appropriate search strategies were developed around radiotherapy, pathways, and simulation. The search criteria used for PubMed were as follows:

(“radiotherapy” OR “radiation oncology”) AND (“waiting list” OR “waiting time” OR “throughput” OR “resource planning”) AND (“simulation” OR “DES” OR “modelling”)

### 2.2 Eligibility criteria

To be eligible for inclusion, the articles had to model multiple sequential processes in the radiotherapy pathway concurrently to demonstrate the suitability of simulation modeling in typical pathway processes. Articles will be included regardless of which aspect of the radiotherapy pathway they model. Papers solely modeling scheduling, capacity, or queuing strategies were excluded.

### 2.3 Study selection and quality

Duplicate studies were removed, and the titles of the remaining articles were assessed for eligibility. Two authors independently screened the abstracts of all studies from the initial search to select articles for data extraction based on inclusion criteria. The quality of studies was assessed by two authors using the reporting checklist developed by Zhang et al.^23^ Any differences were discussed between the two authors to agree on a consensus.

**FIGURE 1:**
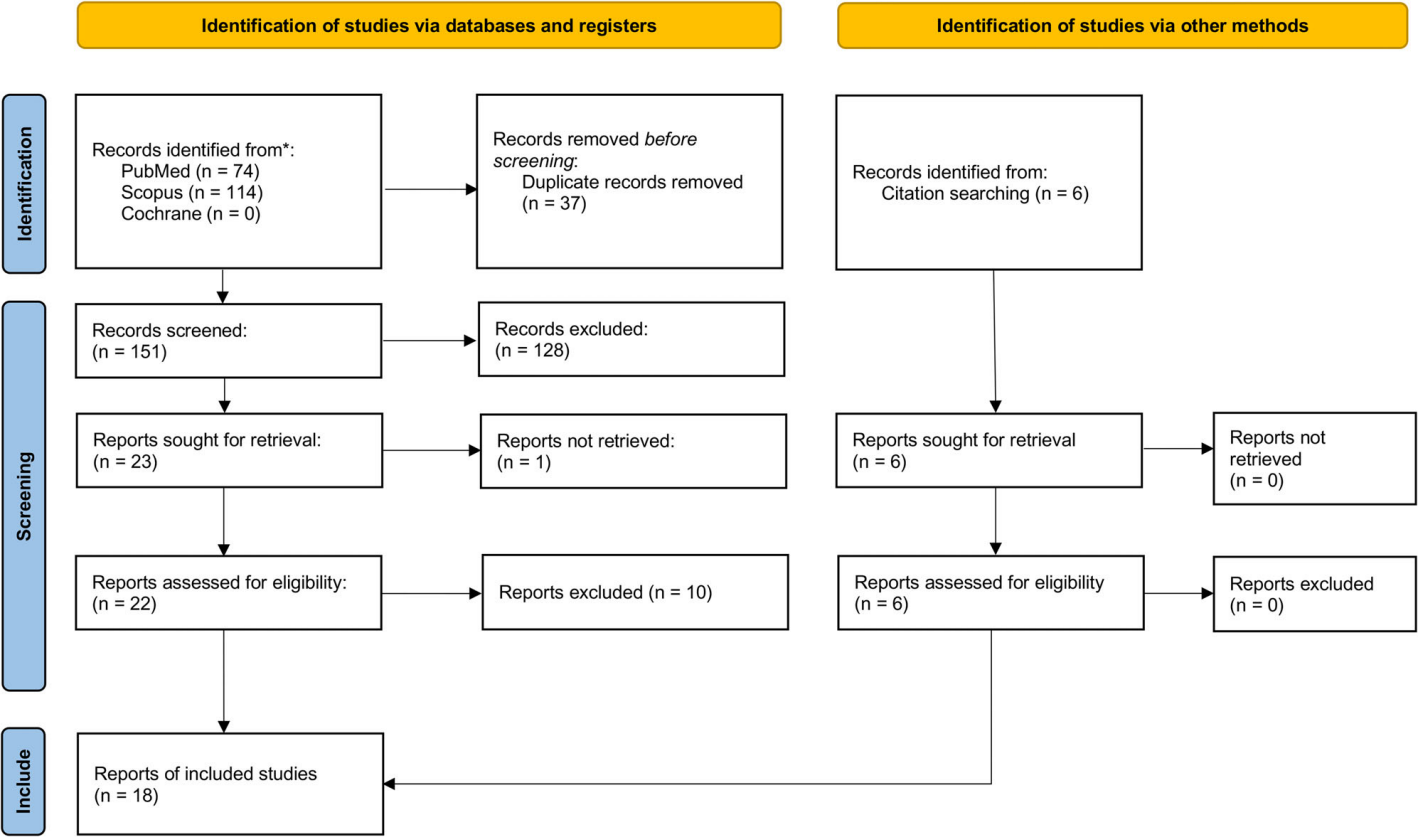
PRISMA flowchart.

Using the Zhang et al. reporting checklist, items were scored as being present or not and expressed as a percentage for each of the four categories (model conceptualization, parameterization and uncertainty assessment, validation, and generalizability and stakeholder involvement) and as a total over the four categories to generate an overall study quality percentage. Due to the binary nature of the scoring, study quality was not used to exclude papers but to indicate an individual paper's compliance with modeling best practices. Figure 1 shows a PRISMA flow diagram and the identification and screening performed.

## 3 RESULTS

### 3.1 Study selection and quality

A PubMed and Scopus databases search yielded 151 articles, and 23 were identified after initial screening. After reference checking, which identified six more studies, and further screening, 18 articles met the eligibility criteria for this systematic review. The study characteristics are shown in Table 1, an assessment of the percentage of studies that addressed specific questions from the Zhang et al. reporting checklist is shown in Table 2, and the details of studies included in this systematic review are shown in Table 3. The average study quality was 63.6% (SD 14.1%). This compares well with Zhang et al., who, over 211 DES simulation studies, found the average rate to be 63.7% (SD 11.0%).

**TABLE 1:** Summary of software used to simulate and pathways modeled.

**Characteristic**	All studies (*N* = 18)
**Software used**	
Arena	4 (22.2 %)
Simul8	3 (16.7 %)
Bespoke/open source	3 (16.7 %)
Other commercial	2 (11.1 %)
FlexSim	2 (11.1 %)
Stella architect	2 (11.1 %)
Spreadsheet	2 (11.1 %)
**Pathway**	
Full	7 (38.8 %)
Pre‐treatment	5 (27.8 %)
Treatment	5 (27.8 %)
Partial pre‐treatment	1 (5.6 %)

**TABLE 2:** Percentage of studies that addressed the quality tools’ questions.

**Model conceptualization**	%
1. Is the focused health‐related decision problem clarified?	100.0
2. Is the modeled healthcare setting/health condition clarified?	100.0
3. Is the model structure described?	88.9
4. Is the time horizon given?	77.8
5. Are all simulated strategies/scenarios specified?	94.4
6. Is the target population described?	94.4
**Parameterization and uncertainty assessment**	
7. Are data sources informing parameter estimations provided?	88.9
8. Are the parameters used to populate model frameworks specified?	66.7
9. Are model uncertainties discussed?	50.0
10. Are sensitivity analyses performed and reported?	33.3
**Validation**	
11. Is face validity evaluated and reported?	66.7
12. Is cross validation performed and reported	0.0
13. Is external validation performed and reported?	5.6
14. Is predictive validation performed or attempted?	66.7
**Generalizability and stakeholder involvement**	
15. Is the model generalizability issue discussed?	44.4
16. Are decision makers or other stakeholders involved in modeling?	66.7
17. Is the source of funding stated?	38.9
18. Are model limitations discussed?	61.1

**TABLE 3:** List of studies and characteristics.

Study	Pathway modelled	Platforms used	Number of patients	Model conceptualisation (%)	Parameterisation and uncertainty assessment (%)	Validation (%)	Generalizability and stakeholder involvement (%)	Overall study quality (%)
Munro et al. (1994) [24]	Pre‐treatment	Spreadsheet	N/A	100.0	75.0	50.0	25.0	66.7
Dickof et al. (2001) [25]	Pre‐treatment	Mathematica	N/A	100.0	75.0	50.0	50.0	72.2
Proctor et al. (2007) [26]	Full pathway	Simul8	N/A	100.0	25.0	50.0	25.0	55.6
Kapamara et al. (2007) [27]	Full pathway	Simul8	Approx. 3000	100.0	50.0	50.0	50.0	66.7
Werker et al.(2009) [17]	Partial pre‐treatment	Arena	10 months’ worth	100.0	100.0	50.0	100.0	88.9
Corazza et al. (2011) [28]	Treatment (protons)	Arena	N/A	83.3	100.0	0.0	25.0	55.6
Joustra et al. (2012) [29]	Full pathway	Medmodel	N/A	66.7	50.0	25.0	25.0	44.4
Aitkenhead et al. (2012) [30]	Treatment (protons)	Matlab/GNU Octave	N/A	100.0	50.0	50.0	50.0	66.7
Crop et al. (2015) [31]	Full pathway	SimPy	N/A	66.7	25.0	0.0	0.0	27.8
Hosseini et al. (2015) [32]	Pre‐treatment (pre‐planning)	FlexSim	N/A	100.0	75.0	50.0	25.0	66.7
Babashov et al. (2017) [33]	Pre‐treatment	Simul8	3888	100.0	75.0	25.0	100.0	77.8
Famiglietti et al (2017) [34]	Treatment (photons)	FlexSim	Approx, 1625	100.0	50.0	25	100	72.2
Vieira et al. (2019) [35]	Pre‐treatment	Tecnomatix	4973	100.0	75.0	50	100	83.3
Saberi et al. (2019) [36]	Full pathway	Arena	5000	83.3	50.0	0	25	44.4
Lindberg et al. (2021) [37]	Full pathway	Stella Architect	3209	100.0	25.0	25	75	61.1
Lindberg et al. (2021) [38]	Full pathway	Stella Architect	3666	83.3	25.0	50	100	66.7
Miranda et al (2021) [39]	Treatment	Arena	233	83.3	75.0	50	50	62.5
Huang et al. (2021) [40]	Treatment	Spreadsheet	N/A	100.0	75.0	25	25	61.1

Across all the studies identified in this review, model conceptualization scored well, with the lowest scoring question being whether the model's time horizon is described (77.8% of studies described this). Validation was the weakest area, with no studies performing true cross‐validation and only one (5.6%) performing external validation. Table two breaks down the overall score for each question across all the studies identified.

### 3.2 Publication data

The first paper identified in this review to model radiotherapy pathways using simulation was published in 1994.^24^ The first study to use commercial software was performed in 2007. Since then, 81% of all simulation studies have used commercial software to perform simulations on radiotherapy pathways.

Most of the research into simulating radiotherapy pathways has been performed in the last 10 years (∼67%). This is probably due to the availability of commercial systems to perform DES to perform simulations, enabling a broader base of users to perform simulations.

### 3.3 Pathways simulated

The following pathways were identified:

- Entire pathway: The pathway from seeing a Radiotherapist/Clinical Oncologist to the end of treatment
- Pre‐treatment: The pathway from seeing Radiotherapist/Clinical Oncologist to starting treatment
- Partial pre‐treatment: The pathway from a point in the pre‐treatment pathway to the beginning treatment
- Treatment workflow: The pathway from a patient entering the department through to the completion of a treatment fraction

Table 1 shows the split of software used to simulate and the pathways modeled from the studies identified. There has been a roughly even split between pre‐treatment and treatment pathway simulations, with pre‐treatment and treatment pathways being subsets of the full pathway, which has been the subject of most research.

### 3.4 Data sources

Oncology Information Systems (OIS) such as Aria and Mosaiq were typically used to acquire the data required to build/verify/validate simulation models. Typical input data needed to be included:

- Cancer category
- Treatment technique
- Dates pathway components were completed

As well as quantitative data from the treatment pathway, qualitative and semi‐quantitative data in staff survey data and interview feedback may have been used. Data describing the arrival process, task times, percentage of re‐work (e.g., replans), and resource availability could also be included.^17^

### 3.5 Motivation for simulation

Although the specifics varied, almost all of the reasons for simulating pathways were to perform sensitivity analysis (e.g., how the system reacts to perturbations) or scenario analysis (e.g., changes to pathways or workflows). The following generic changes to the pathways were modeled:

- Increase/decrease in referrals
- Increase/decrease in staff resource
- Increase/decrease in equipment resource
- Increase/decrease in efficiency
- Changes to schedules
- Machine breakdown
- Severe staffing changes

Regarding output from the simulations, average waiting time was a standard metric and the percentage of patients moving through the pathway in a certain period. The utilization of pathway resources was also a feature of some studies.

## 4 DISCUSSION

This review has identified vital literature for simulating radiotherapy patient pathways. Key themes are discussed below: pathway philosophy, simulation construction, model validation, situations modeled, limitations, and implications for future research.

### 4.1 Pathway philosophy

There are several overarching strategies for scheduling patients, which determine how the pathways are modeled. A pull strategy (also known as a Kanban system) is where the patient's first treatment appointment is scheduled at the beginning of the pathway (typically after their first consultation),^31^ which affords some certainty to the patient about when their treatment will commence. A push strategy is when the patient's first fraction is scheduled after some pre‐treatment activities have been completed. As identified by Vieira et al., where a push strategy is utilized, this can reduce the number of appointments rebooked (e.g., if a task takes longer than expected),^35^ but this may be at the expense of certainty for all parties. Crop et al. simulated a “constant work in progress” system, where new work can only enter the pathway when other work exits the system, the idea being to keep the workload constant (i.e. removing peaks and troughs).^31^ This pathway method increased the number of treatments per day but did not shorten the pathway length.

Radiotherapy departments often use ICD‐10 coding to classify cancer diagnosis.^41^ For each classification used in radiotherapy, there will be at least one radiotherapy pathway. Still, there are likely to be multiple pathways for each ICD‐10 diagnosis that consider clinical staging and other patient‐dependent factors. This can lead to each department having tens, if not hundreds, of pathways. Rather than building every radiotherapy pathway into their simulation, Lindberg et al. performed some novel analysis using the Pareto principle^42^ and grouped pathways by the similarity of resources.^37^ They reduced the number of pathways from 128 to 14/8/21 groups for curative/palliative/no treatment intent using the Pareto method and 7−40/4‐36/7‐82 groups using the grouping strategy, depending on the correlation cut‐off. This demonstrates that simulation performance is not adversely affected by appropriate simplification. The number of pathways was also highlighted by Werker et al., with there being 52 in their study.^17^ It is worth noting that in 2009 when this work was published, the use of IMRT and VMAT techniques for conventional or stereotactic radiotherapy was likely to be less prevalent than today, and that pathway complexity may have increased since then.

One study that had a modest number of pathways was that of Huang et al,^40^ where they modeled 17 pathways, with the four most common pathways being brain CNS, head and neck, prostate, and breast (50 out of 92 cases per day), and 80% of their patients coming from 7 of their 17 (41%) pathways.^40^

### 4.2 Simulation construction

Approximately two‐thirds of the studies used commercial modeling software to perform their simulations. This is unsurprising, as scheduling the radiotherapy process is a complex problem.^43^ This may be because developing in‐house simulations can be time‐consuming and require specialist knowledge that may only be available in some facilities and was explicitly acknowledged by Werker et al.^17^

The commercial systems identified in this review were: Arena, FlexSim, Medmodel, Simul8, Stella Architect, and Tecnomatix. A commercial system has the advantages of manufacturer user support, resources to support inexperienced modelers and a user base that enables collaboration.

The data generated varied from study to study; not all studies reported the number of patients’ records used to create or validate their models. Some studies used less than a year's worth of data. Whilst this might equate to many patients in a large center, some referral patterns might not be captured. The minimum number reported was 233 patients.^39^

In the UK, the modal birth date is the 26th September, corresponding to a peak in an above‐average period of births of approximately 6 weeks (i.e., between the end of August to mid‐October).^44^ If national cancer screening programs are synchronized with birth dates, and these screening programs translate into cancers and radiotherapy referrals, this would require a whole year's worth of data to build a model to minimize the chance of artificial homogeneity being introduced. Additionally, if any process changes are introduced into a pathway during a data collection period, this could introduce undesirable heterogeneities into a model. When looking at seasonal trends, Werker et al. found no significant trends once weekends and holidays were removed.^17^

Some studies included breakdown times.^26^ Breakdowns are inevitable when using complex machinery, and modeling the effect of these on radiotherapy delivery may help develop strategies to minimize disruption.

How patients arrive in the simulations varied slightly from study to study. Proctor et al. had patients arrive in one batch at the beginning of the day, with characteristics such as diagnosis being assigned from probability distributions.^26^ They also utilized a feature of the Simul8 software called “travel time”, which they shortened for urgent cases going through the system. They also assigned priorities for queuing, ranked on the system's characteristics.

Input data for models varied on what aspect of the pathway was being modeled. Still, they tended to include arrival process, tasks times, percentage of rework (if applicable) and resource availability, as identified by Werker et al.^17^ They also highlighted that historical data collected from the center's information systems typically incorporate task time with waiting time in one measure. To overcome this, they included the results from a staff survey to estimate the time taken on tasks. This led to the input to the model assigning tasks times randomly based on uniform distributions.

One factor that models need to incorporate potentially is the skill mix of treatment planning staff; for example, less experienced staff might only be able to work on more straightforward tasks, whereas more experienced staff can perform simple and complex tasks. Werker et al. attributed four staff skill levels. In the UK, as an approximation, this could be translated into pay bands (e.g., 4−7) to account for increased responsibility with more senior staff.

Oncologist availability for their associated tasks will have different complexities. Oncologists in the UK generally treat one or two cancer types, but not all their work time will be dedicated to radiotherapy pathway tasks (such as contouring). In the studies identified, Oncologists are typically modeled in shifts associated with their availability for radiotherapy tasks.^17^

One staff group's availability that may have changed since earlier literature is that of the Medical Physicist. As identified by Werker et al., like Oncologists, Medical Physicists have other responsibilities (i.e. only a proportion of their time is spent on radiotherapy pathway tasks).^17^ Within a Medical Physicist team, some Medical Physicists may devote more time to treatment pathway tasks than others, but this can be modeled by utilizing simulation software shifts and availability patterns.

### 4.3 Validation of models

Face validation was performed in 66.7% of studies. When models were validated, validation methods were broadly similar. A model was created after a run‐up period, its output was run over some time interval (typically 3−6 months), and its output was compared to the real‐world system. A comparison could have been any KPIs of the real‐world system (e.g. time between different points in the pathway).

As highlighted by Werker et al., once a simulation model has been validated, decision‐makers can perform “what if” analyses to examine how their process might react to changes that may be undesirable to perform.^17^ Their validation approach was calculating the average time to create a plan; their model predicted 5.71 days compared to a real‐world 5.67 days. They also looked at the average time for specific plan types, noticing differences of up to 5% between the model and the real world for brain radiotherapy plans.

Vieira et al.’s. model validation compared very favorably to the real system, finding that model versus real system (with 95% confidence interval) waiting time in days was 7.8 (7.5–8.1) versus 7.9 overall, 5.6 (5.4–5.9) versus 5.9 for pull strategies, and 9.7 (9.4–10) versus 9.7 for push strategies.^35^

Babashov et al.’s model validation also demonstrated that their initial model showed similar performance to the real‐world system, finding that model (with 95% confidence interval) versus real system mean waiting time was 10.83 (10.61–11.05) versus 10.82.^33^ Patients meeting the 14‐day waiting time threshold was 82.25% versus 83.59%. The 90th percentile days were 19.87 versus 21. Their warm‐up analysis indicated that the system reached a steady state after 208 days. This is interesting because other studies had varying warm‐up times. For example, Werker et al. used a warm‐up period of five days followed by a run length of 100 days 17, and Proctor et al. had a two‐week warm‐up.^26^

Werker et al.’s approach to validation was to build simple versions in small steps, adding to the model as individual pieces were validated.^17^

To determine variability, models were run multiple times. Proctor et al. ran their model five times, with each run lasting 13‐weeks (only analyzing weeks 3−10 due to warm‐up). Werker et al. utilized 30 replications.^17^ Huang et al. ran 300 simulations for each scenario, although they did not explain their reasoning for doing so.^40^ Warm‐up periods are desirable in simulations to allow a steady state to be reached before starting statistical counters.^45^

Miranda et al. calculated the number of replications required for their model using a method by Law et al.,^45^ requiring 10 for their “exit time of the last patient” (ET) metric and 24 for their “process delay” (PD) metric. In terms of model performance, they found their model generated an ET of 871.55 min versus 869.42 min in the real system, and for their PD metric, 11.47 min for their model and 12.77 min in the real system. No statistically significant difference was found between the results.

### 4.4 Situations modeled

As identified by Vieira et al., discrete event simulation is a powerful tool to model interventions to improve workflows in radiotherapy.^35^ A typical scenario modeled is the introduction of perturbations to the pathway, such as a sudden increase in referrals or unavailability of resources (physical or personnel). In addition to the effects of perturbations on the system regarding waiting times, some studies also built financial analysis in their models. This allowed trade‐offs to be evaluated, such as increased staff costs versus increased income (due to more patients being treated) and whether treating patient types that attract higher revenue (versus an increase in complexity) is worthwhile.

Werker et al. define sensitivity analysis as “varying an input and measuring the extent to which the model outputs are affected”, and the purpose is to “determine for which inputs it is critical to be confident in the accuracy”.^17^ They found that increasing task time has an enormous effect and increases the number of arrivals. They also found that increasing or decreasing oncologist productivity had little effect. They make the distinction between sensitivity analysis and “what‐if” scenarios in that they model “…what would happen to the system if different types of changes were made.”, but stress that results from “what‐if” scenarios should not be taken as fact, but that they may identify scenarios that may be promising to investigate further. One of the main conclusions from their study was that delays associated with oncologists’ tasks were the main impediment to faster planning times.

Lindberg et al. followed up their initial study by modeling different policies to accommodate an interesting facet of Swedish law: employees have a legal right to four successive weeks’ vacation between June and August.^37^, ^38^ They noted, without simulation, that since this occurrence only happens once a year, it might take many years of trial and error to find an optimal strategy to accommodate patients over this period.

Babashov et al.’s modeling showed that their system was susceptible to several scenarios.^33^ One was a staff group with one fewer member (physicist), and the other had a 15% increase in arrivals. The positive change that had the most effect was adding one more dosimetrist to the team (with less than one day's reduction in average waiting time).

Whilst models can identify potential efficiencies, centers must be mindful of the increasing complexity of techniques which can increase the time taken to perform processes, as sometimes the impact of additional complexity can be neglected from the scheduled time allocated to tasks, which increases workload.^46^

As previously identified, approximately 67% of articles were generated in the last 10 years, likely coinciding with the current IGRT and IMRT/VMAT paradigm. However, recent literature is not UK‐based and may not include service techniques like SRS, SABR, etc. Additionally, the UK now has two national proton therapy centers, although earlier modeling by Aitkenhead et al. did model proton radiotherapy treatment processes.^30^

### 4.5 Limitations found

Model limitations were reported in only 61.1% of studies. Simple models, such as early work by Munro et al., could only be used with a single treatment category.^24^ When a simpler treatment completes, such as a palliative, it does not free up all the necessary capacity for a longer or more complicated course of treatment. Munro et al. defined three types of treatment: simple, intermediate, and complex. Each has properties such as the number of fractions, time taken per fraction, available machine hours per week, patients currently waiting, etc.

Werker et al. highlights that models can never perfectly mimic an existing system and that a model might always apply to similar situations without modifications.^17^

Statistician George Box is associated with the aphorism “All models are wrong”,^47^ as any model is only a representation of a real‐world system and will never be entirely correct. This is not to say that models are not useful, something else that Box asserts, and this is why validation of models is such an essential step of any modeling process.

## 5 IMPLICATIONS FOR FUTURE RESEARCH

It was noted that only 28% of studies scored over 70% using a quality assessment tool. This quality assessment tool was created after guidance was issued on modeling good research practices.^48^ Quality assessment tool scores are just one metric to judge a study. However, future modeling studies should consider following published guidance to ensure that studies follow a sound methodology.

No literature was found modeling the introduction of AI‐based auto‐segmentation or knowledge‐based planning (KBP), two tools gaining considerable interest in radiation oncology. What‐if scenarios can model the introduction of processes designed to speed up the pathway's manual or traditionally lengthy processes. Multiple reviews have identified studies utilizing AI‐based auto‐segmentation and KBP, showing potential time savings for these technologies.^49^, ^50^, ^51^ To establish if these technologies' introduction is likely to speed up the overall pathway or generate bottlenecks elsewhere is an area that further modeling research should be undertaken, potentially combined with financial modeling to help judge the cost versus benefit of introducing such systems.

Radiotherapy pathways have likely become too complex for non‐commercial software to perform the simulation unless a group with considerable experience and knowledge in simulation is conducting the research.

As highlighted by other authors, health system professionals can learn significantly from modeling tools to help understand complex dynamic systems.^52^ As highlighted in the pathway philosophy section, researchers need to balance the need for a generalizable model that is not infinitely complex with a model that adequately represents the system they are modeling.

Most studies identified in this review were from European countries (66%), and there is likely to be a significant degree of heterogeneity in the pathway designs employed across all studies (i.e., publicly funded vs. insurance‐based or self‐pay may have different ways of operating, and therefore, pathways). Geographical factors may intrinsically influence how healthcare is accessed, and therefore the pathway, in specific healthcare systems (such as rural communities or accessing national services, such as proton radiotherapy in the UK).

## 6 CONCLUSION

This review has demonstrated that modeling and simulation of radiotherapy pathways are feasible, and those correctly modeled systems generate outputs (such as average waiting times) that match real‐world systems. Validated models give researchers and hospital decision‐makers confidence that they can modify models with potential workflow enhancements to assess their effect and that the model output would represent what would happen in the real‐world system.

Simulations are typically performed to examine the effects of perturbations on average wait times or to assess the percentage of patients starting treatment within a specific period. However, financial modeling can also be built into some models. Financial modeling allows trade‐offs to be assessed, such as increasing staffing to facilitate treating more patients or ensuring that departments can treat referral types with desirable tariffs.

Models cannot easily factor in human nature—some “give and take” might be intrinsically buffered by the system being modeled, for example, by people working additional hours unofficially that are not considered. This may have been exacerbated by increased working from home in the post‐COVID era. Researchers should be mindful of this when making decisions based on simulations. It is recommended that researchers follow best practice guidelines when building models to ensure that they are fit for purpose and to enable decision makers to have confidence in their results.

The introduction of AI‐based auto‐segmentation and KBP, two techniques that reduce the time taken for critical steps in the radiotherapy pathway is an important recent development. The simulation of the introduction of such techniques in the radiotherapy pathway would be helpful to decision‐makers, especially if financial modeling and timesaving are also modeled.

## AUTHOR CONTRIBUTIONS

Conception and design: Andrew Robinson, Md Asaduzzaman, Raj Jena, Roozbeh Naemi. Data collection: Andrew Robinson and Md Asaduzzaman. Analysis and interpretation: Andrew Robinson, Md Asaduzzaman, Raj Jena, Roozbeh Naemi. Draft manuscript preparation: Andrew Robinson, Md Asaduzzaman, Raj Jena, Roozbeh Naemi. All authors reviewed the results and approved the final version of the manuscript.

## CONFLICT OF INTEREST STATEMENT

The authors do not have relevant conflicts of interest to disclose.
